# Narcolepsy and anxiety. Is this association possible?

**DOI:** 10.1192/j.eurpsy.2021.1629

**Published:** 2021-08-13

**Authors:** G.M. Ruiz Martinez, L. Soldado Rodriguez, M. ValverDe Barea

**Affiliations:** 1 Mental Health Unit, Complejo Hospitalario de Jaen, Jaen, Spain; 2 Jaén, Complejo Hospitalario Jaén, Jaén, Spain

**Keywords:** Anxiety, narcolepsy, drowsiness, hypocretin

## Abstract

**Introduction:**

Excessive daytime sleepiness, hypnagogic-hypnopompic hallucinations, sleep paralysis, and cataplexy are symptoms associated with narcolepsy. It is not uncommon to occur co-morbidly between narcolepsy and psychiatric disorders. This association is poorly understood. Recent findings indicate that anxiety disorders also are associated with typical symptoms of narcolepsy.

**Objectives:**

Study of the comorbidity between narcolepsy and psychiatric disorders, like anxiety, through a clinical case.

**Methods:**

A 21-year-old female patient with no psychiatric history who consulted due to anxiety and panic attacks related to poor narcolepsy control. Debut of the neurological disease during adolescence with frequent cataplexy attacks that condition their daily activity and generate avoidance behaviors and agoraphobia.

**Results:**

The patient complained of poor quality of sleep and reported a large number of different types of situations (eg, surprise, embarrassment) associated with cataplectic events. Treatment with SSRIs first and bupropion with pregabalin later was partially effective. Recent studies suggest efficacy of vagus nerve stimulation.

**Conclusions:**

Anxiety disorders, especially panic attacks and social phobias, often affect patients with narcolepsy. Anxiety and mood symptoms could be secondary complications of the chronic symptoms of narcolepsy. Recent studies have shown that narcolepsy is caused by defective hypocretin signaling. As hypocretin neurotransmission is also involved in stress regulation and addiction, this raises the possibility that mood and anxiety symptoms are primary disease phenomena in narcolepsy. Recent studies suggest that vagus nerve stimulation could be potentially useful in the treatment of resistant depressive and anxiety disorder and it is not a contraindication in patients with narcolepsy.

**Disclosure:**

No significant relationships.

